# Drug Resistance and Novel Therapies in Cancers in 2020

**DOI:** 10.3390/cancers15030717

**Published:** 2023-01-24

**Authors:** Zhixiang Wang

**Affiliations:** Department of Medical Genetics, Faculty of Medicine and Dentistry, University of Alberta, Edmonton, AB T6G 2H7, Canada; zwang@ualberta.ca

After a very successful year in 2019 with 34 publications, our Topic collection “Drug Resistance and Novel Therapies in Cancers” guaranteed another productive year with the publication of 17 research articles and 4 review articles in 2020. These research articles covered many different types of cancers, including colorectal cancer (CRC) [1,2,3], prostate cancer (PC) [4,5], epidermal cancer [6,7], bladder cancer [8], gastric cancer (GC) [9], glioma [10,11], lung cancer [2,7], Osteosarcoma (OSA) [12], colitis-associated cancer [13], multiple myeloma [14], ovarian cancer [7], breast cancer [15], and leukaemia [16]. We highlighted a variety of research focusing on the mechanisms of drug action and drug resistance [4,5]. Many studies explored the potential to repurpose existing drugs for application to the treatment of different types of cancers [1,6,7,9]. Some articles were included which examined the effects of various combination therapies on cancer [2,8,12]. Other studies focused on the mechanisms underlying drug action and resistance [3,4,5,10,13,14,15]. Seven of these research articles have been cited on over ten occasions [3,4,6,7,8,11,14]. These research articles were contributed by the leading scientists across the world.

The first research article of the year focused on the effects of sitravatinib, a novel multitargeted receptor tyrosine kinase inhibitor, in reversing multidrug resistance (MDR) [7]. In this drug repurposing research, it was found that sitravatinib blocks the drug efflux function of ABCB1 and ABCG2 in a dose-dependent manner, but does not significantly alter the protein expression of ABCB1 or ABCG2 in multidrug-resistant epidermal cancer cells. The paper has been cited 19 times and is the mostly cited research article from this collection in 2020. Another study aims to understand the mechanisms underlying resistance to cabozantinib, an inhibitor to MET and VEGFR in prostate cancer [4]. The authors identified cabozantinib-induced FGFR1 activation as a novel mechanism conferring cabozantinib resistance. They demonstrate that the molecular basis of resistance to MET inhibition by cabozantinib in prostate cancer is FGFR1 activation through a YAP/TBX5-dependent mechanism. In another study, Wang et al. attempted to overcome cisplatin-resistance by blocking epigenetic process in bladder cancer [8]. The effects of the combination of the DNA methyltransferase inhibitor decitabine (DAC) with the histone deacetylase inhibitor entinostat (ENT) were examined in bladder cancer cells of different platinum sensitivities. Although the combination of DAC and ENT do not directly overcome cisplatin resistance, they are highly toxic to the cancer cells alone. Their data suggest that the combination of DAC and ENT is highly synergistic and has a promising potential for therapeutic applications in bladder cancer, particularly in cases with platinum resistance.

In a search for effective therapeutic agents treating gastric cancer (GC), Ke et al. examine the efficacy of a known cancer drug, tenovin-6, in treating GC [9]. They found that Epstein–Barr virus (EBV)-positive and -negative GC cell lines were sensitive to tenovin-6, albeit at different response times and doses. Tenovin-6 inhibited the anchorage-independent growth of GC cells by inducing apoptosis and cell cycle arrest. They further suggested that treatment with tenovin-6, alone or in combination with chloroquine, an autophagy inhibitor, is a promising therapeutic approach for GC. Similarly, another published piece of research assessed the possibility of using a known cancer drug, 9F, to treat CRC [1]. 9F is a polyamine-vectorized anticancer drug used to treat hepatocellular carcinoma. In this study, the authors demonstrated that 9F inhibits CRC cell growth by inducing apoptosis and cell cycle arrest, and suppresses migration, invasion, and angiogenesis in vitro, resulting in the inhibition of tumor growth and metastasis in vivo. Thus, 9F is a potential therapeutic drug for CRC. Following the identification of autophagonizer (APZ) as an autophagy inhibitor 10 years ago, Kown’s team focused their research on identifying APZ targets and its efficacy in treating glioma [10]. They identified Hsp70 as a key target protein of unmodified APZ in autophagy. Both APZ treatment and Hsp70 inhibition impact the lysosome integrity and lead to autophagic cell death. They demonstrated that APZ can be used as a potent antitumor drug candidate to treat glioma.

Carbon-ion radiotherapy is a highly advanced form radiotherapeutic treatment and is frequently used to treat osteosarcoma (OSA). Although this treatment has yielded promising results, the prognosis of OSA still remains unsatisfactory. In a study published in this collection, scientists from Korea and Japan joined hands to test the efficacy of carbon-ion beam irradiation, both alone and in combination with zoledronic acid (ZOL) on OSA cells [12]. ZOL is widely used in treating bone diseases. They found that this combination greatly inhibited OSA cell motility and invasion. Their results suggest that the carbon-ion beam irradiation, in combination with ZOL, has high potential to increase OSA cell death. A preclinical study focused on the resistance of prostate cancer to enzalutamide (Enz) [5]. Enz is recently developed antiandrogen drug treating prostate cancer. However, despite the drug’s success in prolonging the survival of prostate cancer patients, Enz-resistance inevitably emerges. In this research, the authors found that Enz-resistant prostate cancer (PCa) cells had higher BCL2 expression and examined if targeting BCL2 would influence the Enz sensitivity of PCa. They showed that targeting Enz-induced BCL2 with inhibitor ABT263 could increase Enz sensitivity in both Enz-sensitive and Enz-resistant PCa cells. In another study, a Chinese team investigated the therapeutic roles of glucocorticoids in acute experimental ulcerative colitis and colitis-associated cancer in mice and their immunoregulatory mechanisms [13]. Their findings indicate that glucocorticoid-induced mTOR signaling in epithelial cells is required in the early stages of acute ulcerative colitis as it acts by modulating the dynamics of innate immune cell recruitment and activation.

Elegant research by Czech scientists uncovered novel mechanisms underlying the mechanism of nelfinavir in overcoming resistance to multiple myeloma [14]. Proteasome inhibitors are the major therapeutic drugs for treating multiple myeloma. However, acquired resistance hinders the efficacy of the treatment and results in disease progression or early relapse. Previous work found that some HIV protease inhibitors such as Nelfinavir, an oral anti-HIV drug, overcome the resistance without knowing the underlying mechanisms. In this research, the authors reported that nelfinavir inhibits the TCF11/Nrf1-driven recovery pathway by a dual mode of action. Nelfinavir decreases the total protein level of TCF11/Nrf1 and inhibits TCF11/Nrf1 proteolytic processing, which explains nelfinavir’s effectiveness in the treatment of multiple myeloma. In another attempt to overcome the resistance to TRAIL in lung and CRC cancers, Russian scientists demonstrated that the combination of TRAIL with doxorubicin, bortezomib, and panobinostat dramatically reduced the viability of TRAIL-resistant A549 and HT-29 cells [2].

Another article we promoted aimed to repurposing erdafitinib for the treatment of cancers with multidrug resistance. Erdafitinib is the first fibroblast growth factor receptor (FGFR) kinase inhibitor to be approved by the FDA. They observed that erdafitinib significantly re-sensitizes ABCB1-overexpressing multidrug-resistant cancer cells by inhibiting ABCB1 [7]. Recent evidence suggests that selective targeting of acid-sensing ion channel 1a subunit (ASIC1a) is a perspective strategy for glioma treatment. A new study published in this collection showed that ASIC1a only expresses in U251 MG and A172 glioma cells, but does not display activity in normal astrocytes. It is further revealed that Mambalgin-2, an ASIC1a inhibitor, limits U251 MG and A172 glioma cells growth with EC50 in the nanomolar range without affecting the proliferation of normal astrocytes. These data suggest that mambalgin-2 is a useful hit for the development of new drugs for glioma treatment [11]. Both studies have been cited 15 times.

Cisplatin is a widely used and important anticancer drug. However, it is well-known that cisplatin causes renal salt and water-wasting syndrome (RSWS) as a side effect. The origin of RSWS is obscure. In a study published here, the authors indicate that cisplatin induces RSWS by inhibiting the epithelial Na+ channel [17]. Another CRC research focused on a different hallmark of cancer, altered metabolism of the cell [3]. It is known that L-arginine/nitric oxide pathway metabolites are altered in CRC. An understanding of the underlying mechanisms will provide means for therapeutic intervention. In this published research article, the authors evaluated the underlying changes in pathway enzymes in 55 paired tumor/tumor-adjacent samples and 20 normal mucosa. They found that arginine metabolic pathways were in both tumors and non-transformed mucosa from the tumor vicinity. They also tested the effects of oxicams, a class of non-steroidal anti-inflammatory drugs, on the relevant metabolic pathways. They showed that novel oxicam analogues, equipped with arylpiperazine moiety at the thiazine ring, are much more effective. Targeted endocrine therapy is commonly used to treat estrogen receptor α (ERα)-positive breast cancer. However, the development of resistance poses an urgent clinical problem. In a research article published in this collection, European scientists identified a novel mechanism underlying resistance. Using time-resolved analysis, they illustrated that ATF3, a member of the ATF/CREB family of transcription factors, essential controls the early response to the targeted endocrine therapy by the regulation of MAPK/AKT signaling pathways. Their research suggests that targeting ATF3 and the downstream pathways may overcome the resistance to the targeted endocrine treatment in ERα-positive breast cancer [15]. The last research article published in this collection in 2020 is focused on a novel MDR pathway [16]. Recent studies have associated the Aldo-keto reductase family 1 member C3 (AKR1C3) with the emergence of MDR. In this research article, the authors investigated the ability of Bruton’s tyrosine kinase inhibitors—ibrutinib and acalabrutinib—to block the AKR1C3-mediated inactivation of the anthracycline daunorubicin (Dau). They demonstrated that ibrutinib and acalabrutinib efficiently prevented Dau inactivation when mediated by AKR1C3. Their results suggest that BTK inhibitors could be repurposed to treat leukaemia through the combination of regimens with standard chemotherapeutics, like anthracyclines.

This collection also published four review articles in 2020. One review article was focused on cannabinoids and its application in cancer therapy [18]. Cannabinoids have been the subject of intensive research recently and have garnered tremendous attention. In this review, the authors discussed the most recent findings and compared the properties of various cannabinoids. They thoroughly reviewed the mode of action of cannabinoids and their potential applications in cancer therapy. This review has been highly influential, with 56 citations in just two years. Another review focused on metabolic reprogramming—a cancer hallmark [19]. Targeting metabolic reprogramming is an important strategy in developing cancer therapies. In this review, the authors focused on the emerging mechanisms underlying various aspects of rewired metabolic pathways in terms of cancer chemoresistance. They emphasized the potential of using traditional Chinese medicine as a chemosensitizer for cancer therapy. This review has also been widely cited, with 29 citations so far. In a review discussing the application of electroporation (EP) in the treatment of urological cancers, the authors highlighted the underlying mechanism of EP and discussed the latest progress and development perspectives of EP-based treatments in urology [20]. Upon chemotherapeutic challenges, lysosomes protect the cells by metabolizing the drugs. The last review published in the year discussed the mechanisms underlying the action of classical chemotherapeutic drugs and the adaptive response of the lysosomes. The authors also highlighted the potential to target lysosome function to overcome chemoresistance [21].
